# Large-Scale Preventive Chemotherapy for the Control of Helminth Infection in Western Pacific Countries: Six Years Later

**DOI:** 10.1371/journal.pntd.0000278

**Published:** 2008-08-27

**Authors:** Antonio Montresor, Dai Tran Cong, Mouth Sinuon, Reiko Tsuyuoka, Chitsavang Chanthavisouk, Hanne Strandgaard, Raman Velayudhan, Corinne M. Capuano, Tuan Le Anh, Ah S. Tee Dató

**Affiliations:** 1 World Health Organization, Vietnam Office, Hanoi, Vietnam; 2 National Center for Parasitology, Entomology and Malaria Control, Ministry of Health of Cambodia, Phnom Penh, Cambodia; 3 World Health Organization, Lao People's Democratic Republic Office, Vientiane, Lao People's Democratic Republic; 4 World Health Organization, Philippines Office, Manila, Philippines; 5 World Health Organization, Vanuatu Office, Vanuatu; 6 World Health Organization, Western Pacific Regional Office, Manila, Philippines; London School of Hygiene & Tropical Medicine, United Kingdom

## Abstract

In 2001, Urbani and Palmer published a review of the epidemiological situation of helminthiases in the countries of the Western Pacific Region of the World Health Organization indicating the control needs in the region. Six years after this inspiring article, large-scale preventive chemotherapy for the control of helminthiasis has scaled up dramatically in the region. This paper analyzes the most recent published and unpublished country information on large-scale preventive chemotherapy and summarizes the progress made since 2000. Almost 39 million treatments were provided in 2006 in the region for the control of helminthiasis: nearly 14 million for the control of lymphatic filariasis, more than 22 million for the control of soil-transmitted helminthiasis, and over 2 million for the control of schistosomiasis. In general, control of these helminthiases is progressing well in the Mekong countries and Pacific Islands. In China, despite harboring the majority of the helminth infections of the region, the control activities have not reached the level of coverage of countries with much more limited financial resources. The control of food-borne trematodes is still limited, but pilot activities have been initiated in China, Lao People's Democratic Republic, and Vietnam.

## Introduction

The neglected tropical diseases (NTDs) are a group of parasitic infections that are among the most common infections in the world's poorest populations [1]. Six of the most prevalent NTDs are due to helminths: lymphatic filariasis, soil-transmitted helminthiasis (including ascariasis, trichuriasis, and hookworm infection), schistosomiasis, and food-borne trematode infections. Epidemiological studies suggest extensive geographic overlap among these diseases, especially among impoverished populations with limited access to health services and sanitation [2].

The control of these neglected diseases is considered a crucial step towards achieving the majority of the eight Millennium Development Goals [3], but despite the availability of cost-effective and successful control or elimination interventions, large numbers of the world's poorest individuals remain afflicted with these diseases [4].

The main strategy for controlling these infections, and the main focus of this paper, is the provision of large-scale preventive chemotherapy to the population at risk [5]. Other recommended strategies for the control of most of the helminthiases are the improvement of sanitation and water supply, and provision of health education. The World Health Organization (WHO) recommends, whenever possible, the integration of the control activities for the different NTDs [6] to contain costs and increase efficiency.

In a paper published in 2001, Urbani and Palmer presented the epidemiological situation of helminthiasis in the countries of the Western Pacific Region (WPR) of WHO and recommended an increased commitment for helminth control from their governments and from donors. Since then, an intense advocacy effort has been conducted and several governments, with the support of donor partnerships, integrated the control of the major NTDs into their national health programs [7].

The present review gathers the available data of 2006 from WPR countries (listed in Table 1) on large-scale preventive chemotherapy with a combination of different anthelminthic drugs (listed in Table 2) and compares the present situation with the one six years ago as presented by Urbani and Palmer [8].

**Table 1:** Countries in the Western Pacific Region of WHO and Their Endemicity for Helminthiasis

Country		LF	STH	SCH	FBTs
China			X	X	X
Malaysia		X			
Mekong countries	Cambodia	X	X	X	X
	Lao PDR	X	X	X	X
	Vietnam	X	X		X
Pacific Islands	American Samoa	X	X		
	Cook Islands	X	X		
	Fiji	X	X		
	French Polynesia	X	X		
	Guam		*		
	Kiribati	X	X		
	Marshall Islands	X	X		
	Micronesia, Federated States of	X	X		
	Nauru		X		
	New Caledonia		*		
	Niue	X			
	Northern Mariana Islands, Commonwealth of the				
	Palau		*		
	Papua New Guinea (PNG)	X	X		
	Pitcairn Islands				
	Samoa		*		
	Solomon Islands		X		
	Tokelau		*		
	Tonga	X			
	Tuvalu	X	X		
	Vanuatu	X	X		
	Wallis and Futuna	X	*		
Philippines		X	X	X	X
Non-endemic countries	Australia				
	Brunei Darussalam				
	Japan				
	Korea, Republic of				
	Mongolia				
	New Zealand				
	Singapore				

*: No information available.

FBTs = food-borne trematode infections; LF = lymphatic filariasis; SCH = schistosomiasis; STH = soil–transmitted helminthiasis.

**Table 2:** Drugs Utilized for Preventive Chemotherapy for Different Neglected Diseases Transmitted in WPR Countries

Disease	Parasite	Drugs	Dosage
Lymphatic filariasis	*Wuchereria bancrofti* *Brugia malayi*	DEC **plus** albendazole	6 mg/kg 400 mg
Soil-transmitted helminthiasis	*Ascaris lumbricoides* *Trichuris trichiura* *Ancylostoma duodenale* *Necator americanus*	Mebendazole **or** albendazole	500 mg 400 mg
Schistosomiasis	*Schistosoma japonicum* *Schistosoma mekongi*	Praziquantel	40–60 mg/kg
Food-borne trematode infections	*Opisthorchis viverrini* *Clonorchis sinensis* *Paragonimus* spp.	Praziquantel	40–60 mg/kg

## Materials and Methods

The data presented on the number of people covered by large-scale preventive chemotherapy in the WPR have been gathered from international publications, internal reports from the ministries of health of different countries, technical reports to donors, personal communication to the authors, and direct involvement of the authors.

The coverage of the interventions has been calculated by dividing the number of treated individuals by the populations at risk. These “at risk” populations may be the entire population living in endemic areas where mass drug administration (MDA) interventions are planned or be part of high-risk groups in areas where treatment interventions are targeted.

The data have been presented by disease and by country, then summarized in tables for easy comparison. Some countries (e.g., Lao People's Democratic Republic [PDR] for schistosomiasis) have already provided data referring to 2007; these data are reported but not included in the calculation of the totals that refer only to 2006.

### Lymphatic Filariasis

Lymphatic filariasis (LF) disease is caused by filariae and in WPR countries the main species are *Wuchereria bancrofti* or *Brugia malayi.* The larva is transmitted from human to human by mosquito vectors. Thereafter, the adult worms live in the lymphatic system of humans, where the female produces several thousand larvae (microfilariae). Filarial infection can be asymptomatic or present one or more acute signs (fever, local swelling, pulmonary eosinofilia, and lymphangitis). Chronic complications include lymphoedema, hydrocele, and damage to the kidney and to the lymphatic system [6]. The total number of individuals at risk living in the WPR is over 30 million [9].

#### Lymphatic Filariasis Control Strategy (Table 2)

For this infection, the elimination strategy in WPR countries recommended by WHO is of two possible kinds [10]: distribution of di-ethylcarbamazine-citrate (DEC)-fortified cooking salt to the entire population of the endemic area for one or two years, or MDA with an annual single dose of a combination of two drugs (DEC plus albendazole) administered for five or six consecutive years to the entire eligible population living in the endemic area.

Additional interventions to prevent disability, and in particular skin infection, are also part of the elimination programme and include education on regular washing, skin care, and timely use of antibiotics. The Global Alliance to Eliminate Lymphatic Filariasis (GAELF) is coordinating, supporting, and supervising the Global Programme for the Elimination of Lymphatic Filariasis (PELF) in different endemic countries.

#### Country Activities for Lymphatic Filariasis Control (Table 3)

China launched a large-scale control activity based on the administration of DEC-fortified salt in 1956 [11]. No cases of microfilaremia are currently reported. In 2006, WHO declared China as one of the first countries where lymphatic filariasis was eliminated as a public health problem; hence, no further MDA is necessary.

**Table 3:** Number of Individuals at Risk for Diseases Requiring Large-Scale Preventive Chemotherapy in the WHO Region of the Western Pacific (WPR) in 2006

	Lymphatic Filariasis		Soil-Transmitted Helminthiasis						Schistosomiasis		Food-Borne Trematodiasis	
Target	Entire Population in Endemic Areas		Preschool Children in Endemic Areas		Schoolchildren in Endemic Areas		Women of Child-Bearing Age		Entire Population in Endemic Areas		Entire Population in Endemic Areas	
	At Risk (000)	Treated (%)	At Risk (000)	Treated (%)	At Risk (000)	Treated (%)	At Risk (000)	Treated (%)	At Risk (000)	Treated (%)	At Risk (000)	Treated (%)
China	Eliminated	Not needed	18,200^a^	200 (1%)	33,800^a^	200 (0.5%)	78,000^a^	400 (0.5%)	3,000	2,000 (66%)	24,700	
Malaysia	1,170	834 (71%)	b		b		b		b		b	
Mekong countries												
Cambodia	437	343 (78%)	1,750	1 300 (74%)	2,800	2,775 (98%)^c^	5,900	0	80	80 (100%)		
Lao PDR	11	11^d^	625	344 (55%)	1,000	990 (99%)^c^	2,100	0	80	74^d^	1,897	620^d^
Vietnam	675	599 (88%)	5,000	1 000 (20%)	8,000	5,820 (72%)	16,750	48 (0.2%)	b	—	175	10 (5%)
Pacific Islands												
PNG, MI, FSM	4,266	591 (14%)	970	0	1,574	0	3,289	0	b	—		
Other Pacific Islands	2,285	965 (42%)	21	0	34	0	71	0	b	—		
Philippines	21,275	10 174 (47%)	11,633	6,100 (52%)	18,650	4,000 (21%)	39,000	0	6 700	0	e	
**Total**	**30,121**	**13,509 (45%)**	**38,199**	**8,944 (23%)**	**65,858**	**13,785 (20%)**	**145,110**	**448 (0.3%)**	**9,860**	**2,154 (21%)**	**26,772**	**10 (0.03%)**
Receiving albendazole through PELF or praziquantel through schistosomiasis control		NA		2,800		3,500		4,200		NA		74
Total treated		13,509 (45%)		11,744 (30%)		17,285 (26%)		4,648 (3%)		2,154 (21%)		84 (0.3%)

Data are in thousands.

a According to MoH China, the total number of individuals at risk of STH is 130 million, downsizing the previous estimation of de Silva [14] based on the 1990 survey.

b The disease prevalence is not transmitted or under the threshold requiring large-scale preventive chemotherapy.

c Global target of 75% reached.

d Activities conducted after 2006 and not included in the total.

e Reports of transmission of the disease available in the country but no estimation of the population at risk available.

NA, not applicable.

In Malaysia, the fourth round of MDA was conducted in 2006 with a target of 1.17 million individuals. In the Mekong countries, Cambodia started the MDA programme of Elimination of Lymphatic Filariasis in 2005. In total, 20 districts in four provinces (Rattanakiri, Stung Treng, Siem Reap, and Preah Vihear) are covered by MDA. The intervention targets a population of 437,000 individuals [12].

In Lao PDR, because four cases positive for microfilaremia had been identified in a survey conducted in 2007, an MDA covering over 10,000 individuals living in Phouvong District was planned and started in 2008 (Chitsavang Chanthavisouk, personal communication).

In Vietnam, 675,000 individuals living in six districts at risk (Red River Delta and Quang Binh province) are targeted by the programme [12].

Of the 14 Pacific countries where LF is endemic, 11 completed in 2006 the fifth round of MDA. (American Samoa, Cook Islands, Fiji, French Polynesia, Kiribati, Niue, Samoa, Tonga, Tuvalu, Vanuatu, and Wallis and Fortuna). The total population at risk was 2.28 million [13]. In the remaining three endemic countries of the area, Papua New Guinea, Marshall Islands, and Federates States of Micronesia, with an eligible population of over 4.2 million individuals, between one and three MDA rounds have been administered [13].

The Philippines has the largest population at risk in the region: 40 of the 79 provinces in the country are in need of MDA, with a total at-risk population of approximately 21 million individuals. MDA activities started in 2002; in 2006 approximately 10.2 million received MDA [12]. The Department of Health is planning to target the entire at-risk population in 2008. The LF elimination programme and soil–transmitted helminthiasis control have been integrated since 2006.

### Soil-Transmitted Helminthiasis

Soil-transmitted helminthiases (STHs) are caused by *Ascaris lumbricoides* (roundworm), *Ancylostoma duodenale* and *Necator americanus* (hookworms), and *Trichuris trichiura* (whipworm). This group of infections is highly prevalent in the population of many tropical countries due to climatic conditions favourable for transmission and to the lack of proper sanitation [5].

STH produces a wide range of symptoms that include intestinal manifestations (diarrhea, abdominal pain), general malaise, and weakness that may affect working and learning capacities, and impair physical growth. Hookworms cause chronic intestinal blood loss that frequently results in anemia [6]. The total number of individuals in the WPR that are at risk is approximately 230 million as estimated by de Silva et al. [14] and updated by recent data from China [15].

#### Soil-Transmitted Helminthiasis Control Strategy

The control strategy in the endemic areas for STH consists of regular large-scale preventive chemotherapy with anthelminthic (mebendazole or albendazole) to the three groups at risk (preschool children, schoolchildren, and women of child-bearing age); other recommended interventions are the improvement of sanitation, clean water supplies, and provision of health education. [6]. The Partnership for Parasite Control (PPC) is coordinating STH control activities in the different countries [16].

#### Country Activities for STH Control (Table 3)

In China, the second national survey [15] estimated 130 million individuals infected with STH. The estimated number is significantly lower than the one estimated in the previous survey of 1990 [17]; this is probably due to an improvement in living conditions and sanitation in several provinces of China. Despite this progress, there are 11 provinces in which large-scale preventive chemotherapy in high-risk groups is still necessary to control morbidity [15].

In 2004, pilot control activities (with albendazole) started in five counties, adding up in 2006 to 800,000 at-risk individuals treated). In 2007 an additional eight counties were included, for a total coverage of 3 million people. (These data are not summed in the total because intervention occurred after 2006). According to the national control strategy for helminth control, large-scale preventive chemotherapy is planed and implemented by provincial staff in all the areas where the infection rate of STH is over 30%.

In Malaysia, the prevalence of STH is estimated to be lower than 10% and does not require large-scale drug distribution [18].

**Figure 1:**
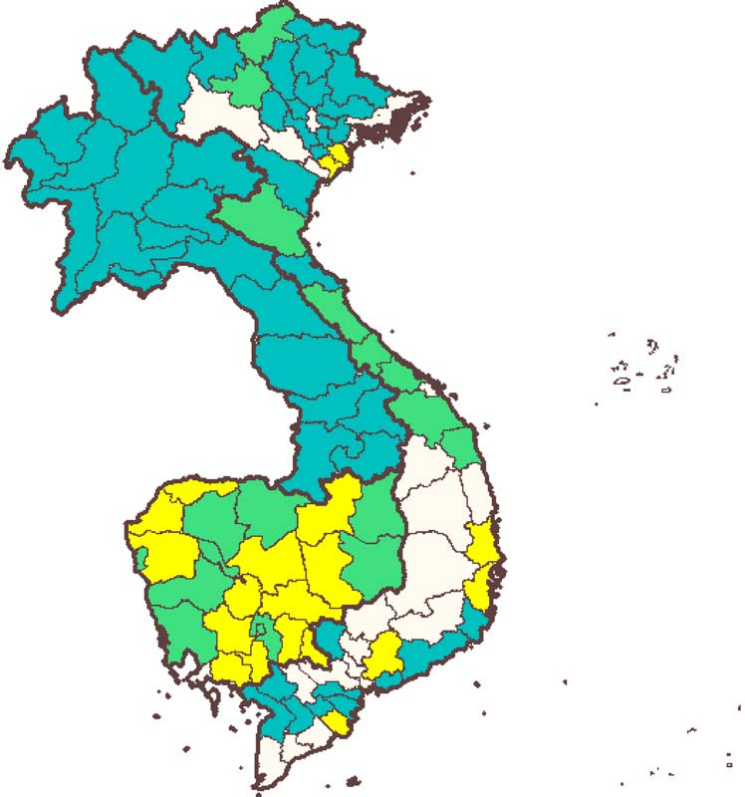
Scaling Up of Preventive Chemotherapy for Schoolchildren in Mekong Countries between 2002 and 2006. Provinces in which all primary schools were covered by the deworming intervention in 2002 are indicated in yellow, the additional provinces covered in 2004 in green, and the additional provinces covered in 2006 in blue.

Since 2002, the Mekong countries have undergone a rapid scale up of the programmes targeting STH in primary schoolchildren (Figure 1). The total number of schoolchildren treated every year in the area increased from approximately 700,000 in 2002 to 9.6 million in 2006 [19],[20],[21]. Cambodia and Lao PDR reached national coverage, respectively, in 2004 and 2007, and Vietnam is expected to reach the target in 2008.

Moreover, approximately 2.6 million preschool children received the deworming treatment during the vitamin A distribution (Mouth Sinuon and Antonio Montresor, personal communication) [21]. Additionally, 48,000 women of child-bearing age (the third group at risk) are regularly treated in Vietnam.

In the Pacific Islands, as expected, the annual distribution of albendazole (in the context of the MDA for the PELF) has significantly lowered the prevalence of STH as in other countries using this anthelminthic combination [22]. In 2007, large-scale preventive chemotherapy with albendazole was conducted in Fiji, Solomon, Tuvalu, and Vanuatu in order to maintain the health gains obtained with the albendazole distributed during the PELF of previous years.

In the Philippines, STHs are widely transmitted [23],[24],[25]. Approximately 10 million children are dewormed every year in the Philippines as part of the mass distribution of albendazole included in the PELF, in the school-based distribution and in a special programme targeting preschool children. In order to reach the WHO target of 75% coverage, an additional 11 million children should be treated in areas not covered by the PELF, but financial resources are not available at the moment.

### Schistosomiasis

The causal agents of schistosomiasis in WPR countries are *Schistosoma japonicum* and *S. mekongi*. The eggs are shed into the environment from the feces of infected individuals. They then hatch in water and liberate the larvae (miracidia) that penetrate the freshwater snail host. After several weeks, cercariae emerge from the snail and penetrate the human skin during water contact. Thereafter, the adult worms deposit the schistosome eggs in the blood vessels surrounding the intestines and cause the disease.

Clinical manifestations are nonspecific (abdominal pain, diarrhea, and blood in the stools). Liver enlargement is common in advanced cases and is frequently associated with ascites and other signs of increased portal pressure [6]. The total number of individuals at risk, living in the WPR, is 10 million [26].

#### Schistosomiasis Control Strategy

The control strategy used in WPR for the control of this infection is the universal treatment with praziquantel administered to the population in the endemic areas [6] (Table 1). Other recommended interventions are the improvement of sanitation, clean water supplies, and the provision of health education [6]. The PPC is coordinating schistosomiasis control activities in the different WPR countries [16].

#### Country Activities for Schistosomiasis Control (Table 3)

The Ministry of Health (MoH) in China embarked on a 10-year (2004–2015) project for schistosomiasis control in provinces located in the lake regions (Anhui, Hubei, Hunan, Jiangsu, and Jiangxi) and in the mountainous regions (Sichuan and Yunnan). Universal treatment with praziquantel is delivered through the primary health care system and is provided free of charge to the populations at risk. A population living in villages where the infection rate is over 10% is considered at risk [27],[28]; 1,334 villages are considered at risk for an estimated population of 3 million [29]. In the lake region, control activities associate distribution of praziquantel [15],[30] with replacement of buffaloes with tractors for agricultural activities, fencing in of buffaloes to avoid pasturing in snail-ridden marshland, improvement of sanitation and water supply, and health education. The total number of individuals treated is approximately 2 million every year [29],[31].

Schistosomiasis is transmitted in four areas in the Philippines (Samar, Leyte, Luzon, and Mindanao). The total population at risk is estimated at 6.7 million [32]. No large-scale preventive chemotherapy is in place at this time.

Within the Mekong countries, schistosomiasis is not transmitted in Vietnam. In Cambodia, large-scale preventive chemotherapy for the control of schistosomiasis covers approximately 80,000 individuals every year in the two endemic provinces of Kratie and Stung Treng; after eight years of large-scale preventive chemotherapy to the entire population, no new cases have been reported since 2006 [33]. Praziquantel distribution intervention is still taking place, but the MoH is planning to increase the interval between two campaigns from one year to two years. In Lao PDR, a similar programme started in 2007 targeting 80,000 individuals in the endemic districts in Champasak province.

In Malaysia and the Pacific Islands, schistosomiasis is not transmitted [26].

### Food-Borne Trematode Infections

This group of diseases is caused by different trematodes. The most common food-borne trematode infections (FBTs) in WPR countries are *Clonorchis sinensis*, *Opistorchis viverrini*, and *Paragonimus* spp. [34].

The eggs leave the human body in feces or in sputum. When in the water, the eggs are directly eaten by the snails or hatch and liberate the larvae (miracidia) that penetrate into freshwater snails. Development in the snail results in the release of numerous cercariae that swim in water until they are eaten by snails, where encystment occurs to form metacercariae. The transmission of FBTs in WPR countries is almost entirely due to the consumption of raw food containing infective metacercariae [34].

Clinical manifestation includes [34]: for clonorchiasis and opistorchiasis: anorexia, abdominal pain, diarrhea, gastrointestinal bleeding, weakness, and weight loss. Clonorchiasis and opistorchiasis are associated with the development of cholangiocarcinoma, and for paragonimiasis, clinical manifestation includes chest pain, cough with rust-colored sputum, fever, fatigue, and fibrotic encapsulation in the lung parenchyma. The total population at risk for FBTs in WPR countries is close to 27 million [34].

#### FBT Control Strategy

Large-scale preventive chemotherapy with praziquantel is the recommended WHO strategy for the control of opistorchiasis, clonorchiasis, and paragonimiasis [34] (Table 1).

#### Country Activities for FBT Control (Table 3)

Despite the fact that a considerable number of individuals are considered at risk of FBT infection in the region, large-scale control interventions have not yet started. In 2005, China initiated control activities in two counties, and in 2008, Lao PDR is planning to start large-scale control activities against opistorchiasis in Champasak province covering 600,000 individuals. In Vietnam, the MoH is conducting a pilot intervention for the control of paragonimiasis in Yen Bai and Lao Cai provinces, and for the control of clonorchiasis in Ninh Binh province, approximately 10,000 at-risk individuals have been treated since 2006. (Antonio Montresor, personal communication).

In Malaysia and the Pacific Islands, FBTs are not transmitted.

### Integration of Treatment

In our opinion, the utilization of the existing government structures and personnel for drug distribution is the most productive way to reduce costs and to increase the efficiency of the large-scale drug interventions. All of the WPR countries have applied this measure. In addition, other forms of integration have been implemented in various WPR countries to further increase efficiency (see Table 4), including

- arranging the timing of drug distribution for diseases addressed with the same drug to comply with the recommended treatment interval for the different diseases,
- providing simultaneously two drugs to the same group at risk of two different parasites, and
- utilizing existing infrastructures to reach specific risk groups.

**Table 4:** Different Kinds of Integration for the Control of NTDs Is Occurring in the WPR

Integration		Intervention 1	Intervention 2	How?	Where?
Two different preventive chemotherapy interventions are integrated	Type 1 (same anthelminthic)	Albendazole (for LF)	Albendazole or mebendazole (for STH)	The same drug is included in preventive chemotherapy interventions against two different diseases. The timing of the drug distributions is arranged in a way that complies with the recommended interval of re-treatment for both diseases.	Cambodia Laos Pacific Islands Philippines Vietnam
		Praziquantel (for schistosomiasis)	Praziquantel (for food-borne trematode infections)		Laos
	Type 2 (different anthelminthics)	Albendazole or mebendazole (for STH)	Praziquantel (for schistosomiasis)		Cambodia Laos Philippines
Drug distribution is integrated within existing infrastructures already reaching groups at risk.	Type 3 (drugs and different intervention)	Albendazole or mebendazole (for STH)	Vitamin A distribution campaigns	The group at risk targeted by preventive chemotherapy interventions and by another programme. The two interventions are co-administered, reducing the need of personnel and transport.	Cambodia Laos Philippines Vietnam
		Albendazole or mebendazole (for STH)	School activities		
					

## Discussion

The elimination of LF is progressively scaling up in the region and it is presently covering 45% of the target population. The Mekong countries and most of the Pacific Islands are conducting efficient programmes. A major benefit of the PELF is the large number of individuals for which STH morbidity is prevented by the albendazole that is part of the combination therapy for LF. Therefore, an important aspect to be considered once the PELF campaigns terminate is how to maintain the benefit of control of STH obtained by the distribution of albendazole.

Large-scale preventive chemotherapy to control STH in schools has begun in all WPR endemic countries, although control activities in China show some delay in scaling up. Activities covering preschool children are in place at significant levels of coverage in the Mekong countries but not in the rest of the region. Despite the significant nutritional improvements by the treatment of women of child-bearing age obtained in Lao PDR [21], the activities for this risk-group are still at pilot level; probably the major impediment for the scaling up of large-scale preventive chemotherapy in women of child-bearing age is the concern of medical personnel about the possible side effects of anthelminthics on unrecognized pregnancies.

China is addressing schistosomiasis as a public health priority; the disease is under control in Cambodia, and a similar control has started in the bordering provinces of Lao PDR. Currently, only the Philippines is late in scaling up the preventive chemotherapy for the control of schistosomiasis.

Preventive chemotherapy campaigns for the control of FBTs have only started in Lao PDR, China, and Vietnam and are at a pilot level. However, a significant scaling up is needed to reduce the morbidity caused by this group of parasites.

In conclusion, six years after the review by Urbani and Palmer [8], a significant step forward has been accomplished in the control of human helminthiasis in most of the WPR countries, demonstrating that, if political commitment exists, even extremely poor countries like Cambodia can reach a significant level of control. However, the majority of the individuals at risk in WPR countries are still in need of appropriate interventions. Therefore, in our opinion, the recommendations made by Urbani and Palmer in 2001 [8] are still valid to promote additional efforts for adequate NTD control.

## References

[1] Hotez PJ, Molyneux DH, Fenwick A, Kumaresan J, Sachs SE (2007). Control of neglected tropical diseases.. N Engl J Med.

[2] Sachs JD, Hotez PJ (2006). Fighting tropical diseases.. Science.

[3] Fenwick A, Molyneux D, Nantulya V (2005). Achieving the Millennium Development Goals.. Lancet.

[4] Molyneux DH (2004). “Neglected” diseases but unrecognised successes—challenges and opportunities for infectious disease control.. Lancet.

[5] WHO (2002). Prevention and control of schistosomiasis and soil-transmitted helminthiasis. Report of a WHO Expert Committee. WHO technical report series: 912. Geneva: World Health Organization.. http://whqlibdoc.who.int/trs/WHO_TRS_912.pdf.

[6] WHO (2006). Preventive chemotherapy in human helminthiasis. Coordinated use of anthelminthic drugs in control interventions: a manual for health professionals and programme managers. Geneva: WHO.. http://whqlibdoc.who.int/publications/2006/9241547103_eng.pdf.

[7] Hotez PJ, Raff S, Fenwick A, Richards F, Molyneux DH (2007). Recent progress in integrated neglected tropical disease control.. Trends Parasitol.

[8] Urbani C, Palmer K (2001). Drug based helminth control in western pacific countries; a general perspective.. Trop Med Int Health.

[9] WHO (2006). The global programme for eliminating lymphatic filariasis.. Wkly Epidemiol Rec.

[10] WHO (2005). Monitoring and epidemiological assessment of the programme to eliminate lymphatic filariasis at implementation unit level..

[11] WHO (2003). Control of lymphatic filariasis in China..

[12] WHO (2007).

[13] WHO (2006). The PacELF way toward the elimination of lymphatic filariasis from the Pacific..

[14] de Silva NR, Brooker S, Hotez PJ, Montresor A, Engels D (2003). Soil-transmitted helminth infections: updating the global picture.. Trends Parasitol.

[15] Ministry of Health China (2005).

[16] Crompton DW, Montresor A, Engels D, Neira M, Savioli L (2003). Action starts now to control disease due to schistosomiasis and soil-transmitted helminthiasis.. Acta Trop.

[17] Xi Long-Qi, Hu Sen-Hai, Xu Shu-Hui, Ministry of Health China (1999). Distribution and Pathogenic impact of human parasites in China [in Chinese]..

[18] Jamaiah I, Rohela M (2005). Prevalence of intestinal parasites among members of the public in Kuala Lumpur, Malaysia.. Southeast Asian J Trop Med Public Health.

[19] Sinuon M, Tsuyuok R, Socheat D, Montresor A, Palmer K (2005). Financial costs of deworming children in all primary schools in Cambodia.. Trans R Soc Trop Med Hyg.

[20] Montresor A, Cong DT, Anh TL, Ehrhardt A, Thach CT (2007). Cost containment in school-deworming programme targeting over 2.7 million children in Vietnam.. Trans R Soc Trop Med Hyg.

[21] Phommasack B, Saklokham K, Chanthavisouk C, Nakhonesid-Fish V, Strandgaard H (2008). Coverage and costs of a school deworming programme in 2007 targeting all primary schools in Lao PDR.. Trans R Soc Trop Med Hyg..

[22] de Silva R, Pathmeswaran A, Fernando SD, Weerasinghe CR, Selvaratnam RR (2003). Impact of mass chemotherapy filariasis control programme on soil-transmitted helminth infections in Sri Lanka.. Ann Trop Med Parasitol.

[23] Lee KJ, Ahn YK, Yong TS (2000). A small-scale survey of intestinal parasite infections among children and adolescents in Legaspi city, the Philippines.. Korean J Parasitol.

[24] Kim BJ, Ock MS, Chung DI, Yong TS, Lee KJ (2003). The intestinal parasite infection status of inhabitants in the Roxas city, The Philippines.. Korean J Parasitol.

[25] Baldo ET, Belizario VY, De Leon WU, Kong HH, Chung DI (2004). Infection status of intestinal parasites in children living in residential institutions in Metro Manila, the Philippines.. Korean J Parasitol.

[26] Engels D, Chitsulo L, Montresor A, Savioli L (2002). The global epidemiological situation of schistosomiasis and new approaches to control and research.. Acta Trop.

[27] Qing-Wu J, Li-Ying W, Jia-Gang G, Ming-Gang C, Xiao-Nong Z (2002). Morbidity control of schistosomiasis in China.. Acta Trop.

[28] Lin DD, Hu GH, Zhang SJ (2005). Optimal combined approaches of field intervention for schistosomiasis control in China.. Acta Trop.

[29] Zhou XN, Guo JG, Wu XH, Jiang QW, Zheng J, Dang H (2007). Epidemiology of schistosomiasis in the People's Republic of China, 2004.. Emerg Infect Dis.

[30] Utzinger J, Zhou XN, Chen MG, Bergquist R (2005). Conquering schistosomiasis in China: the long march.. Acta Trop.

[31] Ministry of Health China (2005).

[32] Leonardo LR, Acosta LP, Olveda RM, Aligui GD (2002). Difficulties and strategies in the control of schistosomiasis in the Philippines.. Acta Trop.

[33] Sinuon M, Tsuyuoka R, Socheat D, Odermatt P, Ohmae H (2006). Control of *Schistosoma mekongi* in Cambodia. Results of eight years of control activities in the two endemic provinces.. Trans R Soc Trop Med Hyg.

[34] WHO (2004). Report of the joint WHO/FAO workshop on food trematode infection in Asia. Report series number RS/2002/GE/40 (VTN).. http://whqlibdoc.who.int/wpro/2004/RS_2002_GE_40(VTN).pdf.

